# The alveolar epithelial differentiation of glandular inner lining cells in a mucoepidermoid carcinoma of the lung: a case report

**DOI:** 10.1186/1746-1596-7-137

**Published:** 2012-10-08

**Authors:** Hong-Tao Xu, Xu-Yong Lin, Qing-Chang Li, En-Hua Wang

**Affiliations:** 1 Department of Pathology, the First Affiliated Hospital and College of Basic Medical Sciences of China Medical University, Shenyang, 110001, China

**Keywords:** Mucoepidermoid carcinoma, Lung neoplasm, Thyroid transcription factor-1, Surfactant protein-B

## Abstract

Mucoepidermoid carcinoma is a common malignant epithelial tumor of salivary glands, but relatively rare in lung. The histological features of mucoepidermoid carcinoma of the lung are similar to its counterpart arising from the salivary glands. Here, we reported a special tumor that occurred in the medial segment of the right lower lobe in a 22-year-old man. This tumor exhibited typical features of mucoepidermoid carcinoma with 3 cell types: squamoid cells, mucin-secreting cells and cells of intermediate type. These 3 types of cells organized into cysts, nests, glands and solid patterns. Specially, the inner lining cells of some glandular structures were uniform cuboidal and hobnail-like, similar to the alveolar epithelial cells. Immunohistochemistry staining revealed that the inner lining cells of glandular structures were positive for thyroid transcription factor-1 and surfactant protein-B, used as markers of alveolar epithelial cells, and were negative for p63. These findings for the first time demonstrated a rare alveolar epithelial differentiation of glandular inner lining cells in a mucoepidermoid carcinoma of the lung.

**Virtual Slides:**

The virtual slide(s) for this article can be found here: http://www.diagnosticpathology.diagnomx.eu/vs/7095988968057804

## Background

Mucoepidermoid carcinoma is a malignant epithelial tumor characterized by the presence of squamoid cells, mucin-secreting cells and cells of intermediate type [1]. It usually arises in the parotid and submandibular salivary glands and in the minor salivary glands of the oral cavity and perimaxillary region. However, mucoepidermoid carcinomas of the lung are relatively rare and comprise less than 1% of all lung tumors. They occur in patients with a wide age range from 3 to 78 years, but 50% of tumors occur in individuals less than 30 years [1-3]. Recently, most cases were generated from the pediatric population [2,4-6]. The majority of mucoepidermoid carcinomas arise from bronchial glands in the central airways. It has been presumed that mucoepidermoid carcinoma is derived from primitive cells within the tracheobronchial mucous glands. Histologically and immunohistochemically, Mucoepidermoid carcinoma of the lung is similar to its counterpart arising from the salivary glands [1,2]. Recent cytogenetic analysis of mucoepidermoid carcinomas using comparative genomic hybridization and spectral karyotyping showed multiple reciprocal translocations. The mucoepidermoid carcinoma translocated 1-mastermind-like 2 (MECT1/MAML2) translocation can be demonstrated in large proportion of mucoepidermoid carcinomas [2,7]. Here, to the best of our knowledge, we for the first time reported the alveolar epithelial differentiation of glandular inner lining cells in a mucoepidermoid carcinoma of the lung. 

**Figure 1:**
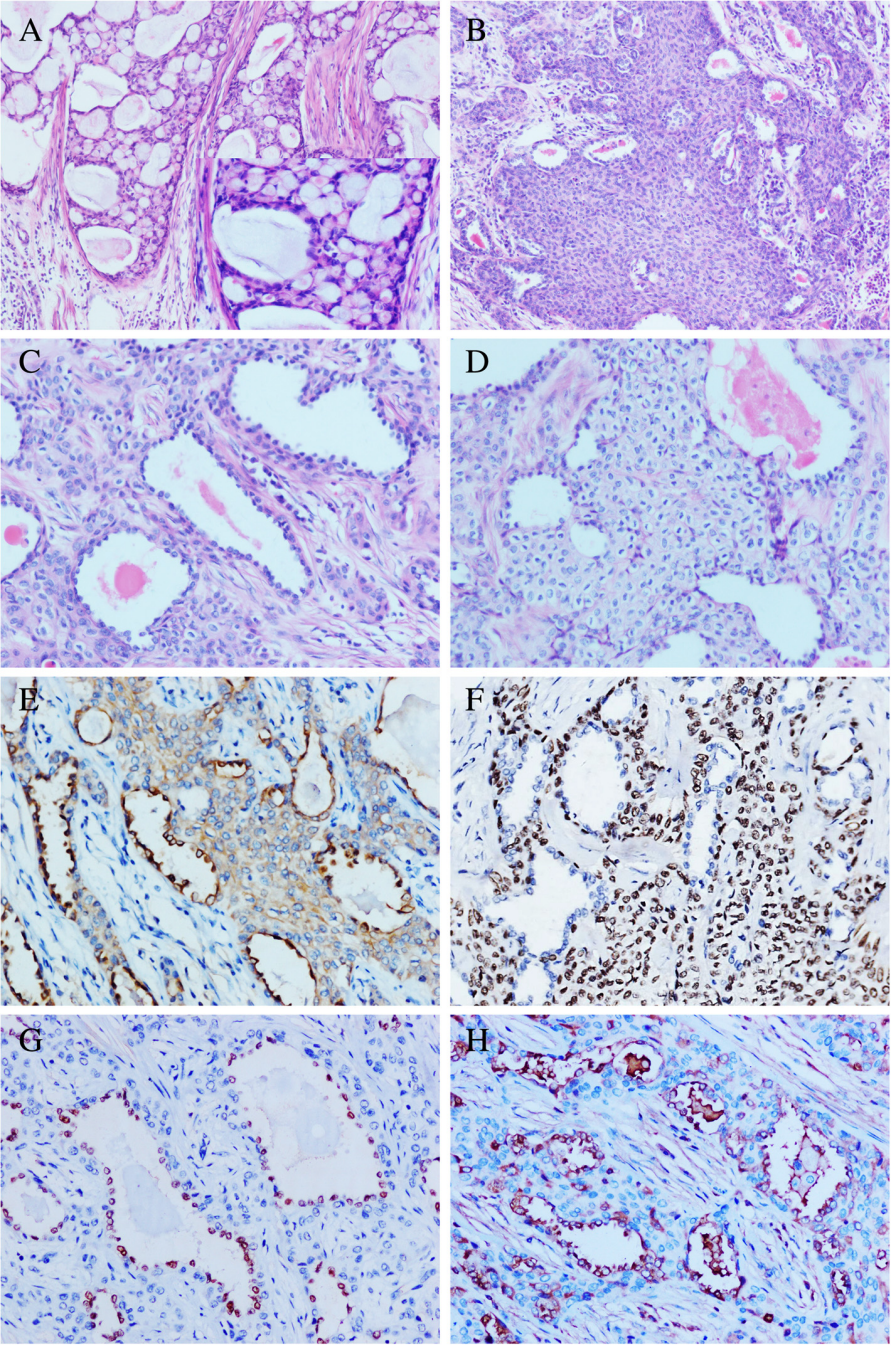
**The histological features and immunohistochemical staining patterns of the present mucoepidermoid carcinoma of the lung. A**: The cysts of the present tumor were variable sizes and contained plentiful mucus. The mucus-secreting cells admixed among the cysts and nests regions were large and had light blue-gray mucinous cytoplasm (H&E ×200). The right bottom is a focal high power field of figure A (H&E ×400). **B**: The glands and nests regions were composed by the intermediate cells, which were polygonal shape, had round bland nuclei and faint eosinophilic cytoplasm (H&E ×200). **C**: The inner lining cells of the glandular structures were uniform cuboidal and hobnail-like, had oval and bland nuclei, similar to the Type II alveolar epithelia (H&E ×400). **D**: The squamoid cells admixed with intermediate cells and formed some nests in a sheet-like pattern (H&E ×400). **E**: Low-molecular-weitht cytokeratin was strongly positive in all tumor cells (×400). **F**: P63 was strongly positive in most of the tumor cells except of the inner lining cells of glandular structures (×400). **G**: Thyroid transcription factor-1 was only limited to the inner layer cells of the glandular structures (×400). **H**: Surfactant protein-B was strongly positive in the inner layer cells and some outer layer cells of glandular structures (×400).

## Case presentation

### Clinical history

A 22-year-old Chinese man presented with a 4.5 cm nodular mass in the medial segment of the right lower lobe. Computed tomography revealed a well-circumscribed mass with bronchi involvement. The bronchus of right lower lobe was truncated. The distal lung was atelectasis. Bilateral hilar did not increase and no enlarged lymph node was found in the mediastinum. The tumor was resected by lobectomy.

### Gross features

Gross pathologic examination revealed that the tumor was round, well-circumscribed, about 4.5 cm in diameter and very close to the bronchus. The cut surface of the tumor was gray-white and brittle texture without hemorrhagic or necrotic foci.

### Microscopic features

The resected tumor was fixed with 10% neutral-buffered formalin and embedded in paraffin blocks. Tissue blocks were cut into 4-μm sections. The histological evaluation was performed using hematoxylin and eosin stained sections. This tumor displayed typical features of mucoepidermoid carcinoma with 3 cell types: squamoid cells, mucin-secreting cells and cells of intermediate type. These 3 types of cells organized into cysts, nests, glands and solid patterns. The cysts were variable sizes and contained plentiful mucus with a colloid-like appearance. The mucus-secreting cells, which admixed among the cysts and nests regions, were large and had light blue-gray mucinous cytoplasm (Figure 1A). The intermediate cells were polygonal shape with round bland nuclei and faint eosinophilic cytoplasm. The intermediate cells were located at the periphery of the glands or formed nests (Figure 1B). The glandular structures were composed of inner lining layer of epithelial cells surrounded by outer layer intermediate cells. Interestingly, in addition to the 3 cell types, this tumor displayed “the fourth cell type”. The inner lining cells were uniform cuboidal and hobnail-like at some regions. These cells had oval and bland nuclei, and were similar to the Type II alveolar epithelia (Figure 1C). Some of the glandular structures contained eosinophilic contents. The squamoid cells admixed with intermediate cells and formed some nests in a sheet-like pattern (Figure 1D). The nuclear mitoses were very few. The calcification or necrotic foci were not observed.

### Immunohistochemistry

The sections were immunostained with primary antibodies against high- and low-molecular-weight cytokeratins, p63, Î±-smooth muscle actin (Î±-SMA), thyroid transcription factor-1 (TTF-1), surfactant protein-B (SP-B), synaptophysin and Ki67. All antibodies were purchased from Maixin, Fuzhou, China. After incubation with primary antibodies, the detection of antibodies was accomplished using the streptavidin-peroxidase method.

Immunohistochemically, high- and low-molecular-weight cytokeratins were strongly positive in almost all tumor cells (Figure 1E). P63 was strongly positive in most tumor cells except of the inner lining cells of the glandular components (Figure 1F). α-SMA and synaptophysin were negative. Interestingly, the positive expression of TTF-1 was only limited to the inner layer cells of the glandular structures (Figure 1G). Furthermore, SP-B was strongly positive in the inner layer cells and some outer layer cells of the glandular structures (Figure 1H). The positive rate of Ki67 was about 3%.

## Discussion

Based on the histological features and the immunohistochemical staining profiles described above, the present lung tumor was diagnosed as a low-grade mucoepidermoid carcinoma of the lung. Mucoepidermoid carcinomas are malignant tumors, but they usually have an indolent behavior. Low-grade mucoepidermoid carcinomas have a much better prognosis than high-grade carcinomas. Mucoepidermoid carcinomas of the lung are often treated by lobectomy, sleeve resection, local resection, segmental resection, or even endoscopic removal [1,2,5,6,8]. Although some studies indicated the lymph node metastasis [6,8], the metastasis of low-grade mucoepidermoid carcinomas of lung are rare [1].

The present tumor was composed of typical three cell types. The mucin-secreting cells were located in the cysts and nests regions, had light blue-gray mucinous cytoplasm, and were positive for cytokeratins staining. The majority of intermediate cells formed nests and solid regions, and were positive for cytokeratins and p63 staining. The intermediate cells also could be found at the periphery of the glandular structures. The squamoid cells formed small nests among intermediate cells, and were positive for cytokeratins and p63 staining. The tumor cells were relatively uniform, and had bland nuclei. The nuclear mitoses were very few. No individual cell keratinization or squamous pearl was observed. So, the diagnosis of adenosquamous carcinoma could easily be excluded. The negative expression of synaptophysin indicated this tumor had not neuroendocrine differentiation. All these evidences supported the diagnosis of a mucoepidermoid carcinoma of the lung.

Interestingly, the appearance of inner lining cells of the glandular structures was different to the typical three cell types. The inner lining cells were cuboidal and hobnail-like, had oval and bland nuclei. We hypothesized that the inner lining cells might be alveolar-epithelial-like cells and examined the expressions of TTF-1 and SP-B. Alveolar epithelial cells express TTF-1 and surfactant proteins, including SP-A, SP-B, SP-C, and SP-D, which are considered as the markers of pulmonary epithelial differentiation and are useful parts of immunohistochemical panel in pulmonary pathology [9,10]. TTF-1, a homeodomain nuclear transcription protein, is considered a specific marker of epithelial cells of the thyroid and lung. SP-B is synthesized and secreted primarily by alveolar type II epithelial cells and essential for normal lung surfactant function. The immunohistochemical staining revealed that the inner lining cells were positive for cytokeratins, TTF-1, and SP-B, but negative for p63. We considered that the inner lining cells might represent focal tumor cells differentiating toward alveolar epithelial cells or entrapped alveolar epithelial cells. But, this primary tumor of bronchus is 4.5 cm in diameter. The alveolar-epithelial-like inner lining cells could be found in more than 1/3 area of the tumor, including the glandular region and the center of some nests or solid regions. Furthermore, the inner lining cells were not only located at the edge of the tumor, but also could be found in the center region of the tumor. Taken together, we considered that these inner lining cells were tumor cells differentiating toward alveolar epithelial cells.

To the best of our knowledge, the alveolar epithelial differentiation was not reported previously in mucoepidermoid carcinomas of the lung. But, it was documented in other salivary gland tumors of the lung, such as salivary gland-type mixed tumors and epithelial-myoepithelial tumors [11-13]. The TTF-1 positive tumor cells were also located at the inner layers of glandular components in these tumors, and were considered as alveolar epithelial differentiation [11-13]. It is indicated that the alveolar epithelial differentiation of glandular epithelia might be a special character in the salivary gland tumors of the lung and should be noted.

Previous studies examined the expression of TTF-1 in a small numbers of mucoepidermoid carcinomas of the lung. They showed that all mucoepidermoid carcinomas of the bronchus in their series were negative for TTF-1 [2,14]. So, it was considered that TTF-1 was useful in differentiating mucoepidermoid carcinoma of the bronchus from primary lung adenosquamous carcinoma [2]. Here, we reported a special and rare case of mucoepidermoid carcinoma with TTF-1 and SP-B positive glandular epithelial cells, which required attention in differential diagnosis. It was reported that the expression of TTF-1 was negative in the mucoepidermoid carcinomas of sinonasal tract (19 cases) [15], But was positive in the mucoepidermoid carcinomas of thyroid gland (2 of 4 cases) [16]. So, we considered that only detecting the expression of TTF-1 was not enough for differential diagnosis. TTF-1 together with SP-B might be useful in distinguishing between primary mucoepidermoid carcinomas of the lung and metastatic tumors from other regions. More cases need to be examined to confirm this hypothesis.

## Conclusions

We for the first time demonstrated a rare morphology of the mucoepidermoid carcinoma of the lung. In this case, the inner lining cells of glandular structures were cuboidal and hobnail-like. They were positive for TTF-1 and SP-B, but negative for p63. We concluded that the inner lining cells of glandular structures were differentiated toward alveolar epithelium, which would bring us new understanding about mucoepidermoid carcinomas of the lung.

## Consent

Written informed consent was obtained from the parents of the patient for publication of this case report and accompanying images. A copy of the written consent is available for review by the Editor-in Chief of this Journal.

## Competing interests

The authors declare that they have no competing interests.

## Authors’ contributions

XHT examined the tissue sections, analyzed the data and wrote the manuscript as a major contributor. LXY helped to collect the clinical data and performed the immunohistochemical analysis. LQC and WEH helped to revise the discussion section of this manuscript. All authors have read and approved the final manuscript.
